# Understanding psychotrauma in Sub-Saharan Africa: a scoping review of clinical and sociocultural perspectives

**DOI:** 10.3389/fpsyg.2025.1606131

**Published:** 2025-12-19

**Authors:** Justin Cikuru, Philippe Kaganda, Adélaïde Blavier, Jennifer Foucart

**Affiliations:** 1 Health Psychology and Human Motricity, Université Libre de Bruxelles, Brussels, Belgium; 2 Social Sciences, Université Evangélique en Afrique, Bukavu, Democratic Republic of Congo; 3 Center of Expertise in Psychotrauma and Legal Psychology, University of Liège, Liège, Belgium

**Keywords:** PTSD, Sub-Saharan Africa, war trauma, sexual violence, culture, expression trauma

## Abstract

**Introduction:**

Since independence, African states have faced armed conflicts and economic crises, disrupting traditional cultures and causing trauma. In Sub-Saharan Africa, psycho trauma is seen as a shared expression of distress rather than a medical condition. Understanding these cultural manifestations is crucial for evaluating Diagnostic and Statistical Manual of Mental Disorders (DSM-5) Posttraumatic stress disorder (PTSD) criteria. This review explores how PTSD symptoms manifest differently in these communities, aiming to enhance cultural understanding and improve patient care.

**Methods:**

We conducted a double bind scoping review following the PRISMA guidelines. A systematic search was performed across multiple databases, including ScienceDirect, Cairn.info, SAGE Journals, Google Scholar, APA PsycInfo, Springer Link, PubMed, and ProQuest, for publications from 2016 to 2024 using the keywords PTSD, Sub-Saharan Africa, war trauma, sexual violence, culture, and expression of trauma.

**Results:**

The search returned 268 records. We identified 15 studies, written in English and French, that met the inclusion criteria. The studies were conducted in 8 countries Burkina Faso (*n* = 1) Cameroon (*n* = 2), Democratic Republic of the Congo (*n* = 4), Ivory coast (2), Kenya (1), Republic of Congo *n* = 1), South Africa (3) and Togo (*n* = 1). Nine studies (60%) highlight the symptoms related to PTSD showing that the DSM-5 emphasis psychological dimensions, whereas in sub-Sahara PTSD is primarily attributed to spiritual causes. Six studies (40%) highlight the meaning of symptoms in African anthropological structures, and the challenges faced by clinical psychologists in the context where mental health is not integrated in the cultural model of healing which involves spiritual rituals, traditional healers, and religious interventions.

**Conclusion:**

This scoping review underscores the necessity of a comprehensive understanding of PTSD in Sub-Saharan Africa, where trauma is predominantly expressed through somatic and spiritual frameworks. Recognizing these cultural expressions is essential for developing contextually relevant diagnostic approaches and improving culturally adapted patient care.

## 1 Introduction

In the last two decades, various armed conflicts have been reported worldwide, but the largest number of conflicts and potential warring situations are currently on the African continent (Hortence, 2022).

Posttraumatic stress disorder (PTSD) is one of the most studied psychological consequences of violence and conflict, yet its manifestations and interpretations are strongly shaped by cultural and contextual factors (Forkus et al., 2023). The Diagnostic and Statistical Manual of Mental Disorders (DSM-5) definition of PTSD, widely applied in psychiatry and clinical psychology, has been criticized for neglecting cultural and linguistic influences on trauma experiences, distress narratives, and healing processes (Al Joboory et al., 2019; Morrison and Dwarika, 2022; Nguimfack and Mbarga, 2021). In Sub-Saharan Africa, trauma responses often rely on cultural knowledge, distress idioms, rituals, and social practices that provide shared vocabularies for expressing suffering and mobilizing social support (Dye, 2018; Bovey et al., 2024; Koudou et al., 2016; Levy, 2021). Understanding these culturally embedded expressions is essential for evaluating whether Western diagnostic frameworks, especially the DSM-5, adequately capture lived experiences of trauma in African contexts.

Armed conflicts remain widespread in Sub-Saharan Africa, driven by territorial disputes, ethnic tensions, political struggles, and economic claims over valuable resources (Leader Maynard, 2019; Yamulamba and Akinola, 2024; Hatem et al., 2025) causing violence-related injuries (Kuupiel et al., 2024). Countries such as the Democratic Republic of the Congo (DRC), Somalia, Sudan, Mali, and Burkina Faso rank among the least peaceful globally (Thiede et al., 2020; Hortence, 2022). These conflicts have devastating social consequences, including displacement, poverty, and breakdown of traditional support systems (Bendavid et al., 2021; Omotoso et al., 2023). In eastern DRC, for instance, prolonged violence has deeply disrupted the social and economic fabric of communities, with psycho-traumatic effects reported in over 80% of the population (Maheshe et al., 2023).

The psychotrauma is defined as the overwhelming of an individual's coping mechanisms following traumatic events (Hudgins and Durost, 2022; Ogden et al., 2021). While not all trauma exposure results in PTSD, around 10–20% of affected individuals may develop the disorder depending on protective and risk factors (Houllé et al., 2017; Luciano et al., 2022; Watkins et al., 2018). PTSD symptoms often include intrusions, dissociation, hypervigilance, or somatic expressions such as rapid heartbeat or sweating (Fraysse et al., 2020; Yuan et al., 2021). However, trauma-related disorders extend beyond PTSD to encompass depression, anxiety, sleep disturbances, and substance use (Ciccone et al., 2020; Diamond et al., 2022; Xiao et al., 2022).

These symptoms depend on pre-traumatic factors (e.g., psychiatric history, personal resilience), peri-traumatic factors (e.g., intensity and nature of the event), and post-event factors (e.g., social support, medical care; Luciano et al., 2022. Furthermore, the onset of psychopathologies may be immediate, delayed, or resolve over time (Diamond et al., 2022). Individual traits (e.g., gender, personal resources) and contextual elements (e.g., social support) significantly shape trauma responses (Xiao et al., 2022). Trauma-related disorders extend beyond PTSD, encompassing various conditions requiring a nuanced understanding for proper diagnosis and treatment. These include depressive, anxiety, sleep, and substance use disorders, alongside broader psychological, somatic, and socio-cultural suffering (Ciccone et al., 2020). Forkus et al. (2023) emphasize the need for trauma conceptualization to account for individuals' environments and cultural contexts, yet such perspectives are not fully addressed in the DSM-5.

In many Sub-Saharan African contexts, psychotrauma is not primarily conceptualized as a medical disorder but as a culturally meaningful way of expressing distress and mobilizing community-based healing resources (Levy, 2021). This raises critical questions about the universality of DSM-5 categories and the need for culturally sensitive diagnostic and therapeutic approaches. Research suggests that trauma responses vary across cultures and that language and cultural norms shape what is perceived as traumatic (Bokanowski, 2021). Individuals in prolonged conflict situations use cultural knowledge and distress idioms to interpret and cope with violence (Dye, 2018; Bovey et al., 2024). Rituals, symbols, and beliefs further help individuals process trauma and communicate suffering within their communities (Bovey et al., 2024; Koudou et al., 2016).

Understanding these cultural manifestations is essential for assessing the relevance of DSM-5 diagnostic criteria for PTSD in war-affected populations. Our approach seeks to interrogate how Western diagnostic frameworks intersect with African cultural understandings, rather than imposing them as universal categories. The present scoping review, therefore, aims to synthesize existing literature on cultural interpretations and expressions of PTSD symptoms in Sub-Saharan Africa. Specifically, it examines how trauma is manifested, described, and managed in local contexts; how these expressions converge or diverge from DSM-5 definitions; and what implications arise for culturally informed trauma care.

## 2 Methods

This scoping review was guided by the Joanna Briggs Institute (JBI) methodology (Hadie, 2024) and reported according to Preferred Reporting Items for Systematic reviews and Meta-Analyses extension for Scoping Reviews (PRISMA-ScR) checklist and explanation (Tricco et al., 2018). Study selection was conducted independently by two reviewers (J.C. and J.F.) based on titles and abstracts. Discrepancies were resolved through discussion. Full-text articles were then screened against the inclusion and exclusion criteria by the same two reviewers.

### 2.1 Research question

How are the symptoms of post-traumatic stress disorder (PTSD) expressed among Sub-Saharan African populations, and in what ways do these manifestations differ from the diagnostic criteria outlined in the DSM-5?

### 2.2 Identified studies

A systematic literature search was conducted between October 2022 and June 2024 across multiple databases, including PubMed, Google Scholar (used to capture additional regionally accessible sources), APA PsycInfo, Springer Link, SAGE Journals, ScienceDirect, and ProQuest. The search utilized standardized search strings combining keywords such as PTSD, Sub-Saharan Africa, war trauma, sexual violence, anthropology, culture, and expression of trauma. Medical Subject Headings (MeSH) were applied only in PubMed, while equivalent keyword-based strategies were adapted for the other databases. In addition to the database searches, we conducted a hand search of reference lists from included articles and selected journals to identify additional studies that may not have been captured through electronic searches. This approach helps ensure comprehensive coverage of relevant literature, particularly studies published in regionally focused journals or sources with limited indexing. Including this step enhances the rigor and completeness of the scoping review and aligns with best practices recommended for culturally specific research.

### 2.3 Study selection

Publications identified through the search strategy were downloaded and imported into Zotero. Duplicate entries were then removed. Two researchers (JC and JF) independently screened the titles and abstracts of the articles based on pre-defined inclusion and exclusion criteria. Studies were included only when both researchers reached an agreement. Any discrepancies were resolved through consensus. Additionally, JC and JF conducted a full-text review, and any uncertainties regarding eligibility were settled through mutual agreement. This dual independent screening ensured inter-rater reliability and minimized selection bias.

### 2.4 Inclusion and exclusion criteria (PCC)

The selection of studies followed the PCC model (Population, Concept, Context):

- Population: populations of sub-Saharan Africa.
- Concept: expression of symptoms of post-traumatic stress (PTSD).
- Context: exposure to situations of war and associated violence.

#### 2.4.1 Inclusion criteria

The selected articles focused on PTSD or traumatic expression in the context of war or armed conflicts. Trauma was considered in a broad sense, encompassing both physical and psychological consequences of war, including sexual abuse, violence, and rape. Only studies published between 2016 and 2024 were included. Furthermore, the articles had to be either case study analyses or observational studies specifically examining adults in Sub-Saharan Africa. We chose to include case reports (*n* = 1) when they provided rich cultural insights into idioms of distress, recognizing their value for understanding localized symptom expression despite limited generalizability.

#### 2.4.2 Exclusion criteria

Articles were excluded if they did not address the specified topics, focused on children or immigrant populations, given their distinct developmental and contextual characteristics, which merit dedicated reviews beyond the present adult-focused scope, or were not case study analyses or observational studies of adults.

### 2.5 Data charting process and analysis

A data-charting form was designed to extract key variables relevant to the study. These variables included literature characteristics such as the author, year of publication, country, study design, and key themes reflected in the research. Additionally, the form captured the study's aim, a detailed description of PTSD symptoms and their cultural manifestations, as well as a comparative analysis of these symptoms with the diagnostic criteria outlined in the DSM-5.

The analysis focused on identifying recurrent themes across studies and contrasting clinical interpretations with sociocultural explanatory models. JC and JF engaged in iterative discussions to refine emerging categories until a shared consensus was reached.

Finally, a summary of collected data and an explanation of the findings and the meanings attached to them are reported, along with research gaps that should be addressed by future studies, as well as policy and practice implications. To report the results, we used PRISMA Extension for Scoping Reviews (PRISMA-ScR; Tricco et al., 2018).

## 3 Results

### 3.1 Study flow

**Figure 1:**
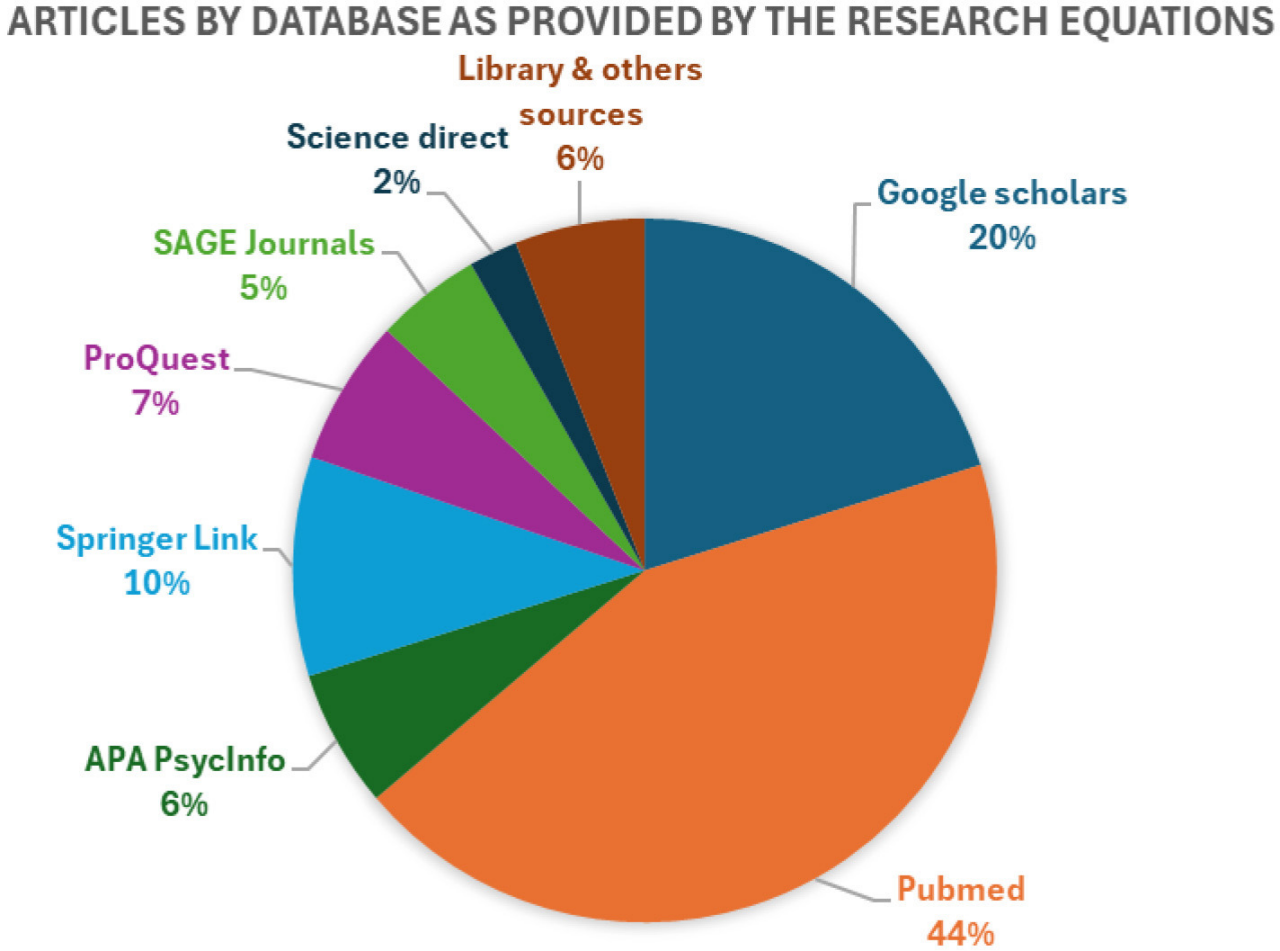
Articles by database as provided by the research equations percentage.

Unlike systematic reviews, our scoping review approach did not aim to synthesize or aggregate findings. Instead, we provide a narrative account structured around thematic analysis to address our research questions. Two search strings were developed. The first was applied in PubMed (*n* = 117) using MeSH terms, as follows: ((((sub Saharan Africa) AND (war trauma OR sexual violence))) AND ((anthropology, cultural[MeSH Terms]) OR (assimilation, cultural[MeSH Terms])) AND (y_10[Filter])) OR ((((culture) OR (customs) AND (y_10[Filter])) AND ((ptsd) OR (TSPT) AND (y_10[Filter]))) AND (expression trauma AND (y_10[Filter])) AND (y_10[Filter])). The second search string was developed using equivalent keyword-based terms, adapted for databases that do not support MeSH. These included Google Scholar (*n* = 54), APA PsycINFO (*n* = 17), Springer Link (*n* = 27), ProQuest (*n* = 18), SAGE Journals (*n* = 13), ScienceDirect (*n* = 6), and Records identified from hand search (*n* = 16) as presented in Figure 1 and Table 1.

**Table 1:** Articles database consulted and geographical distribution.

**Database**	**Total**	**Countries**	**Total**
Pubmed	117	Burkina Faso	1
Google scholars	54	Cameroon	2
Springer Link	27	Democratic Republic of the Congo	4
APA ProQuest	18	Ivory cost	2
PsycInfo	17	Kenya	1
SAGE Journals	13	Republic of Congo	1
Science direct	6	South Africa	3
Library and others sources	16	Togo	1
Total	268	Total	15

### 3.2 Literature searching

**Figure 2:**
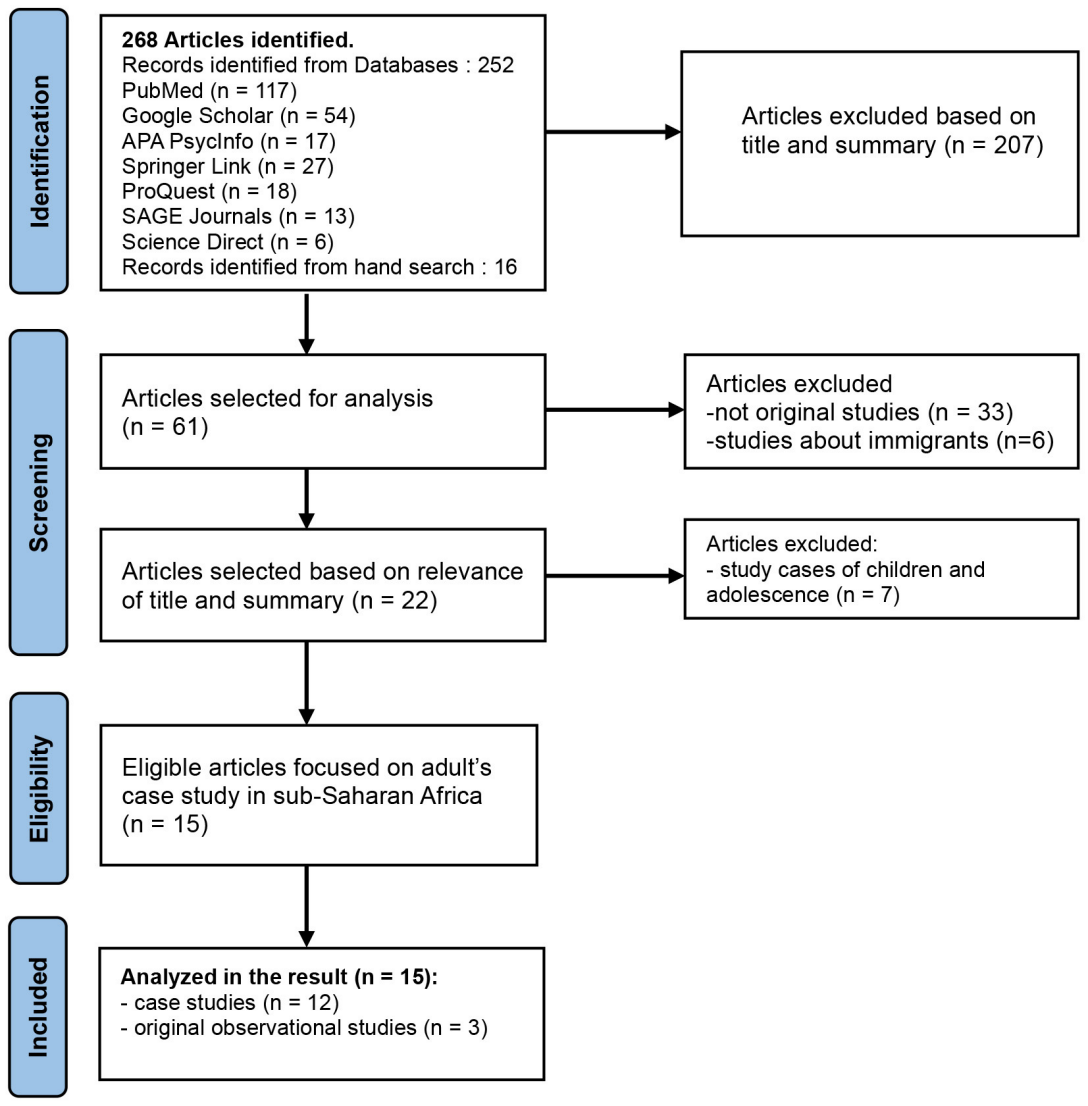
Prisma flow chart of study selection.

We identified 268 articles through search engines and other hand search. We screened titles and abstracts, excluding studies based on title and summary (*n* = 207), those that were not original studies (*n* = 33), those focused solely on immigrants (*n* = 6), and those addressing children/adolescents (*n* = 7), resulting in 15 included studies (see Figure 2). The number of subjects studied in those fifteen studies ranged from 1 to 212 cases. The selected publications were mainly conducted in the Burkina Faso (*n* = 1), Cameroon (*n* = 2), Democratic republic of the Congo/DRC (*n* = 4), the Ivory Coast (*n* = 2), Kenya (*n* = 1), the Republic of Congo/Brazzaville (*n* = 1), South Africa (*n* = 3) and Togo (*n* = 1) as presented in Table 1. The analysis of the included studies identified three central thematic domains (Table 2): symptoms presented by individuals who experienced psychotrauma; the cultural and anthropological interpretations of these symptoms in African contexts, and the role of clinical psychologists in Sub-Saharan Africa.

**Table 2:** Data reflecting themes from 15 studies used in the scoping review.

**N°**	**Countries**	**Authors**	**Study design**	**Publication**	**Reflected themes in the studies**
1	Burkina-Faso	Desjardins et al. (2022)	Case study (*n* = 20)	Canadian Journal of Development Studies	The symptoms presented by patients
2	Cameroon	Nguimfack and Mbarga (2020)	Case study (*n* = 4)	Psychologie Clinique	The symptoms presented by patients, The meaning of symptoms according to the African anthropological model, The role of psychologists in the African healthcare system.
3		Nguimfack and Mbarga (2021)	Case study (*n* = 4)	L'information psychiatrique	The symptoms presented by patients, The meaning of symptoms according to the African anthropological model, The role of psychologists in the African healthcare system.
4	Democratic republic of the Congo	Benjamin et al. (2019)	Case study (*n* = 1)	Annales Médico-psychologiques, revue psychiatrique	The meaning of symptoms according to the African anthropological model
5		Maisha et al. (2017)	Observational study (*n* = 15)	Social Sciences	The symptoms presented by patients,
6		Mavinga et al. (2022)	Case study (*n* = 89)	European Scientific Journal	The meaning of symptoms according to the African anthropological model
7		Grevisse et al. (2019)	Case study (*n* = 9)	International Journal of Innovation and Applied Studies	The meaning of symptoms according to the African anthropological model, The role of psychologists in the African healthcare system.
8	Ivory cost	Koudou et al. (2016)	Case study (*n* = 57)	Rivista di Criminologia, Vittimologia e Sicurezza	The meaning of symptoms according to the African anthropological model, The role of psychologists in the African healthcare system
9		Koudou et al. (2019)	Case study (*n* = 212)	Sciences & Actions Sociales	The meaning of symptoms according to the African anthropological model
10	Kenya	Rousseau and Cook-Cottone (2018)	Observational study (*n* = 17)	Complementary Therapies in Medicine	The symptoms presented by patients, The role of psychologists in the African healthcare system.
11	Republic of Congo	Mouanga et al. (2018)	Case study (*n* = 4)	L'information psychiatrique	The symptoms presented by patients, The role of psychologists in the African healthcare system.
12	South Africa	Knettel et al. (2020)	Case study (*n* = 1)	Psychotherapy	The symptoms presented by patients, The meaning of symptoms according to the African anthropological model
13		Kaminer et al. (2018)	Case study (*n* = 3)	Journal of Health Psychology	The symptoms presented by patients
14		Hadie (2024)	Observational study (*n* = 7)	Journal of Child & Adolescent Trauma	The symptoms presented by patients
15	Togo	Adansikou et al. (2017)	Case study (*n* = 144)	Annales Médico-psychologiques, revue psychiatrique	The symptoms presented by patients, The meaning of symptoms according to the African anthropological model

### 3.3 Thematic presentation of PTSD symptoms

#### 3.3.1 Somatic symptoms

Studies exploring symptoms related to PTSD mainly examine victims of sexual violence and war-related torture. These include civilians, prisoners, refugees, and military personnel exposed to traumatic events in sub-Saharan Africa. The symptoms described in this section appeared in nine articles without reference to clinical diagnosis. Observed symptoms included, physical fatigue, sexual disturbances, palpitations, dry mouth, sleep disturbances (Kaminer et al., 2018; Nguimfack and Mbarga, 2021). Another set of symptoms emphasizing somatic expression ranged from unexplained organic diseases to various ailments such as intestinal infections, angina, laryngitis, bronchitis, fevers, abscesses, loss of teeth and hair, headaches, eye disease, loss of smell, rheumatism, torticollis, muscle atrophy, ear whistling, and ear infections (Knettel et al., 2020; Mouanga et al., 2018). The reviewed articles highlight that in Sub-Saharan Africa, these symptoms are often interpreted as witchcraft attacks by an enemy, particularly when physicians find no medical cause (Koudou et al., 2016; Mavinga et al., 2022).

#### 3.3.2 Psychological symptoms

According to Nguimfack and Mbarga (2021), PTSD symptoms in an African sociocultural context must be situated within a context shaped by ancestral beliefs, traditional customs, and ritual practices. These include the use of amulets, fetishes, and communal rituals through which the individual's personality is structured by relational networks. Thus, to understand how mental disorders develop in sub-Saharan African cultural context requires recognizing three constituent dimensions of the individual. These dimensions are defined in three poles according to Nguimfack and Mbarga (2021): the community pole or the domain of maraboutage involving collective allegiance of a clan or tribe to a religious leader or teacher. This leader is expected to provide blessings, consultations, and solutions to family or social problems. the pole of witchcraft, referring to the use of alleged supernatural powers or magic to control people or events. PTSD, therefore, produces a disorder that involves a triple relationship related to the ancestral believe, the sociocultural environment, and the nuclear family (Langevin et al., 2018). In terms of the ancestral dimension, aggression or violence is the work of a person possessed by mystical forces who uses amulets to perform their deeds. The aggressors are perceived as being possessed by a demon that drives them to cause harm, which in turn leads to persecution dreams, social withdrawal, anger outbursts, irritability, and hypervigilance (Desjardins et al., 2022; Koudou et al., 2019; Mavinga et al., 2022; Nguimfack and Mbarga, 2021). In the DRC, for example, traditional culture considers rape a curse that defiles the victim, while the aggressor is believed to be possessed by mystical forces. Thus, in the DRC cultural setting, treating rape and its consequences requires traditional rituals, often conducted by guardians of cultural norms (Maisha et al., 2017).

#### 3.3.3 Spiritual symptoms

The symptoms that reveal spiritual impairment include psychotic decompensation. These manifest as depersonalization, derealization, dream image, hallucinated vision, awareness of mystical dreams, incoherent speech, nudity, and a strong feeling of evil spirit possession. Our scoping review highlights that these symptoms have an anthropological meaning in sub-Saharan Africa and are often interpreted in relation to the ancestor. Thus, psycho-traumatic experiences are understood as conflicts with traditions and community values (Al Joboory et al., 2019; Brohm, 2017; Van der Kolk et al., 2016). Nguimfack and Mbarga (2021) report a clinical case in which a soldier testifies that those who become insane after war are those who refused to go through the traditional rite in the village. In this context, what DSM-5 classifies as PTSD symptoms is understood as punishment. From an anthropological perspective, the soldier's symptoms reflect a blend of traditional and religious interpretations, rather than a psychological one. The symptoms identified across the reviewed studies encompass nearly all psycho-traumatic signs that could lead to a PTSD diagnosis (Mavinga et al., 2022). However, unlike DSM-5 models, the explanatory framework does not emphasize neurobiological, cognitive, or psychoanalytic dimensions. Instead, the symptoms are linked to disruptions in cultural, family, and spiritual elements, whose significance must be understood within the anthropological context in which they arise.

### 3.4 Meaning of PTSD symptoms within African anthropological structures

In sub-Saharan Africa, mental illness and trauma are often conceptualized through communal, spiritual, and ancestral frameworks (Nguimfack and Mbarga, 2021). Sow, as cited by Nguimfack and Mbarga (2021), posits that PTSD manifests as elements of persecutory violence perceived as external to the victim. This perception operates on three interconnected levels: the imaginary, daily reality, and the symbolic world. Individuals may experience a sense of being possessed by the spirits of the deceased, which can often result in extreme behaviors. (Desjardins et al. 2022), (Koudou et al. 2019), (Mouanga et al. 2018), and (Nguimfack and Mbarga 2020) further explain that possession experiences can result in violent outbursts, During these episodes, the individual may have no recollection of their actions. Knettel et al. (2020) highlight that these episodes are often accompanied by acute anxiety attacks, including palpitations, excessive sweating, and trembling. Understanding these culturally specific interpretations is crucial for developing effective mental health interventions in the region.

In many sub-Saharan African cultures, dreams are integral to understanding personal and communal experiences (Koudou et al., 2019). Encounters with ghosts in dreams are common. However dreams that evoke intense emotions, especially linked to persistent psychological trauma, are considered abnormal. These dreams often become focal points for community discussions (Mavinga et al., 2022). Individuals experiencing such distressing dreams frequently seek guidance from cultural authorities or traditional healers (Al Joboory et al., 2019; Maisha et al., 2017). These cultural interpreters may attribute the distress to spiritual disturbances or unresolved ancestral issues. These interpreters may attribute the distress to spiritual disturbances or unresolved ancestral issues. Subsequently, rituals are performed to alleviate suffering and restore harmony (Maisha et al., 2017). This approach contrasts with the DSM-5, which primarily associates recurrent distressing dreams with traumatic events and emphasizes individual psychopathology.

By adopting an anthropological perspective, evil spirit possession is a representation of mental illness in Africa (Adansikou et al., 2017). Indeed, a significant proportion of the population (40%) considers mental illness to be of magico-religious origin (Nguimfack and Mbarga, 2021). PTSD symptoms are therefore interpreted as relational difficulties between the ancestor and the psycho-traumatized individual, often linked to prohibitions or violations of traditional rules (Adansikou et al., 2017; Grevisse et al., 2019). Maisha et al. (2017) emphasize that rape is seen as a profound transgression. It defiles the victim and disrupts their connection with ancestral spirits. Such acts are believed to invite malevolent forces, causing mental disturbances. Victims often experience intense guilt and fear of stigmatization, anticipating ostracism from families and communities. Koudou et al. (2019) report that PTSD symptoms in sub-Saharan Africa include persecution dreams, nightmares, and hallucinations. Any perceived separation from the ancestor may immediately lead to trauma. Treatment often involves consulting a fetishist, a person capable of neutralizing harmful witchcraft. Delusional reconstructions such as nightmares and hallucinations arise when the fetishist identifies the assailant. This power infiltrates the victim's essence through sorcery, threats, and the use of bark, human or animal bones, teeth, and red or black fabrics. Experiences such as torture, violence, and other potentially traumatic events are often perceived as integral aspects of life rather than isolated incidents (Mouanga et al., 2018; Nguimfack and Mbarga, 2021).

This perspective is deeply rooted in the communal and spiritual fabric of sub-Saharan societies. Adversity is viewed as a collective experience requiring communal resilience and support (Knettel et al., 2020). Fear associated with violence, torture, and rape is seen not as a medical condition, but as a state of helplessness or a situation that can cause harm (Adansikou et al., 2017). Bodily injury, risk of disease, or pregnancy are often of greater concern to the victim than the violence itself (Maisha et al., 2017). Trauma is considered pathological only when it leads to biological or physical consequences (Klimcik et al., 2020). Symptoms that the DSM-5 classifies as psychological, such as reliving experiences or persecution dreams, are often managed through prayer or other cultural practices (Bihabwa and Kangami, 2018; Mouanga et al., 2018; Nguimfack and Mbarga, 2021).

In most cases, trauma is shared by the entire family (Maisha et al., 2017). This contradicts the individual-level conception of PTSD in DSM-5. Individuals are not seen as autonomous entities but as having a familial self. Experiences are considered in the intersubjective domain of the group rather than at the individual or intrapsychic level (Adansikou et al., 2017; Bihabwa et al., 2019; Grevisse et al., 2019; Koudou et al., 2016; Maisha et al., 2017; Mouanga et al., 2018). Beloucif (2021) explains that the mind and body are interdependent in Sub-Saharan African culture. Illness is primarily perceived as a physical condition, while the psyche is less recognized as affected. Consequently, victims predominantly express traumatic suffering through somatic symptoms. PTSD is therefore seen less as a psychological diagnosis and more as a condition influenced by spiritual factors (Knettel et al., 2020). Women survivors of sexual or domestic violence often present with internalizing symptoms such as guilt, shame, silence, and social withdrawal, often framed through spiritual or cultural lenses. Men exposed to conflict or displacement may display externalizing symptoms such as aggression, substance use, or behavioral withdrawal (Maisha et al., 2017; Mouanga et al., 2018).

DSM-5 presents victims of traumatic events as passive sufferers at the individual level (Grevisse et al., 2019). In contrast, PTSD in sub-Saharan Africa is understood in relation to external factors, such as angry ancestors or spirits seeking appeasement. Nightmares or paranoia are attributed to witchcraft or spiritual attacks rather than internalized past experiences (Nguimfack and Mbarga, 2021). Experiences of trauma and avoidance behaviors are often interpreted spiritually, linked to supernatural forces (Allon, 2016). While DSM-5 integrates somatic and psychological dimensions, victims in Sub-Saharan Africa primarily attribute trauma to spiritual causes when no physical explanation is found (Grevisse et al., 2019). DSM-5 directs victims to psychologists and psychiatrists (American Psychiatric Association, 2016). Conversely, African societies adopt a holistic perspective, where help is sought from medical doctors, religious leaders such as pastors, or traditional healers like fetish priests (Grevisse et al., 2019; Knettel et al., 2020; Nguimfack and Mbarga, 2021; Womersley and Kloetzer, 2018; Grupp et al., 2018).

### 3.5 The challenge of the clinical psychologist profession in Sub-Saharan Africa

Six studies explore the role of clinical psychologists in Sub-Saharan Africa. Traditional practitioners address PTSD symptoms through witchcraft, community rituals, and prayer as part of the healing process (Allon, 2016; Caizzi and Ciambellini, 2017; Nguimfack and Mbarga, 2020, 2021; Yagi et al., 2022). These symptoms are often interpreted as the effects of a fetish, malevolent magic, or a curse inflicted by an enemy. Alternatively, they may be seen as consequences of cultural transgressions or sins, depending on individual beliefs (Grevisse et al., 2019; Morrison and Dwarika, 2022; Mouanga et al., 2018).

Most victims of rape have reported turning to traditional healers for purification rituals or to receive magic potions for their healing. This practice was a strategy or customary rule for the reintegration of victims into their families and communities. For some couples, even though the husbands were Christians and their faith was crucial in their decision to stay married after a rape, this traditional integration ritual remained essential for husbands to accept their raped wives (Maisha et al., 2017). This situation creates a dilemma for sub-Saharan African clinical psychologists trained in Western models. Victims often expect them to act as healers or intercessors, rather than as mental health professionals who provide support through listening and counseling (Sikkema et al., 2018). Furthermore, the psychologist's role outside medical and spiritual realms is not yet fully recognized. When victims cannot find relief through doctors, traditional practitioners, or pastors, they often turn to God and their ancestors (Klimcik et al., 2020). In sub-Saharan regions drugs are central to medical treatment. Many believe that if medication is unavailable, a condition cannot be cured unless it is spiritual. As we know, psychologists do not provide drugs to the patients, only psychiatrists do, which complicates understanding their role in this context.

Thus, if the medical doctor does not find a medical cause for the victims' symptoms, the victim often searches for the cause in the spiritual world, seeking help from God or ancestors (Caizzi and Ciambellini, 2017; Grevisse et al., 2019; Womersley and Kloetzer, 2018). This belief is established early in life through childhood initiation rites, both traditional and religious starting in the family and continuing in the community. These practices are considered non-negotiable and constitute a dynamic organization of an individual's life within their developmental environment (Mouanga et al., 2018). Therefore, African psychologists must prove that they can bring added value to earn their place in a healing model that differs from the Western-inspired professional training that they have received.

### 3.6 Comparison table of key differences between the DSM-5 framework and the Sub-Saharan African cultural context regarding trauma

The DSM-5 provides a universal, medicalized approach to diagnosing trauma-related disorders (Forkus et al., 2023), in contrast, African cultural contexts interpret PTSD symptoms through traditional and religious beliefs (Sikkema et al., 2018). Table 3 summarizes the key differences between the DSM-5 framework and the Sub-Saharan African cultural context. It shows contrasts in symptom conceptualization, etiology, cultural significance, and treatment strategies across the two contexts. The table illustrates that DSM-5 uses standardized diagnostic criteria, whereas sub-Saharan societies incorporate spiritual, communal, and ritual dimensions in understanding and addressing trauma.

**Table 3:** Key differences between the DSM-5 framework and the Sub-Saharan African cultural context.

**Study themes**	**Items**	**DSM-5 perspective**	**Sub-Saharan African context**
Symptoms related to PTSD	Cultural interpretation of symptoms	Symptoms of trauma-related disorders are viewed through a clinical, psychological lens, categorized based on standardized diagnostic criteria (e.g., intrusive thoughts, avoidance, hyperarousal)	Symptoms may be interpreted as a result of spiritual punishment, ancestral displeasure, or violation of traditional norms rather than a mental health disorder
	Etiology of the symptoms	Trauma symptoms result from exposure to extreme stressors, affecting brain function and emotional regulation	Trauma symptoms may be seen as a consequence of breaking traditional taboos, curses, or supernatural forces (e.g., spirits seeking revenge or punishment for not performing required rituals)
The meaning of symptoms in African anthropological structures	Expression of symptoms	Symptoms are categorized into specific diagnostic criteria (e.g., nightmares, flashbacks, emotional numbness)	Symptoms may manifest as somatic complaints (e.g., body pain, paralysis, fatigue), possession states, or visions/dreams involving ancestors which are not explicitly recognized as PTSD symptoms in the DSM-5
	Social stigma vs. communal support	Mental health disorders can carry stigma, often leading to isolation or reluctance to seek treatment	Trauma symptoms may be understood and addressed within a communal framework, where elders, religious leaders, and family play a central role in healing. However, if perceived as a supernatural curse, individuals may also face rejection
The challenges faced by clinical psychologists in the context	Treatment approaches	Focuses on evidence-based psychological and pharmacological treatments, such as cognitive-behavioral therapy (CBT) and medication	Healing often involves spiritual rituals, traditional healers, and religious interventions (e.g., purification ceremonies, prayers, sacrifices)

## 4 Discussion

This scoping review analyzed fifteen articles, primarily case analyses and observational studies published between 2016 and 2024, exploring the manifestation of PTSD symptoms in Sub-Saharan African communities and their divergence from DSM-5 criteria. Trauma responses in Sub-Saharan African contexts are shaped by cultural, linguistic, and societal frameworks. What may be considered traumatic in Western psychiatric models is often understood differently in Sub-Saharan contexts, where spiritual, communal, and symbolic dimensions play an essential role in defining and addressing psychological suffering (Bokanowski, 2021). It is important to note that this work is a scoping review of academic literature rather than clinical case material; the reflections offered therefore constitute theoretical critiques of diagnostic frameworks, not empirical patient data. Clarifying this distinction ensures that the contribution is properly situated within scholarly debates on trauma and culture.

Beyond describing individual studies, this review identifies three major cross-cutting insights. First, PTSD in Sub-Saharan Africa is often conceptualized as a disruption of social and spiritual harmony rather than as an individual psychopathology. Second, symptom expression tends to emphasize somatic and relational dimensions over the intrusive-reexperiencing model of the DSM-5. Third, healing responses privilege communal and ritual forms of restoration rather than individualized clinical treatment. Together, these patterns highlight both the universality of post-traumatic suffering and the cultural variability in its meanings and treatments.

### 4.1 The cultural interpretation of PTSD symptoms

Understanding PTSD symptoms in Sub-Saharan Africa requires examining them within cultural, familial, gender and spiritual frameworks. Across the reviewed studies, gender consistently emerged as a key social determinant shaping both trauma exposure and interpretation. Female gender emerged as a robust predictor of PTSD (Bihabwa and Kangami, 2018; Di et al., 2022; Dozio et al., 2016; Tesfaye et al., 2024). Similarly, in a high-adversity cohort of South African women, women were more likely than men to exhibit probable PTSD, even when exposure levels and co-occurring depression were controlled (Altare et al., 2020; Hiscox et al., 2021; Langevin et al., 2018; Nguimfack and Mbarga, 2020). Unlike the DSM-5, which defines PTSD through neurobiological and psychological mechanisms, Sub-Saharan African perspectives often link these symptoms to failures in fulfilling traditional rituals and spiritual obligations. PTSD is frequently interpreted through religious and ancestral beliefs rather than psychological paradigms (Nguimfack and Mbarga, 2020).

This divergence illustrates how diagnostic constructs are mediated by local ontologies of personhood, whereas the DSM-5 privileges internal psychological processes, many African frameworks locate suffering in disrupted social or spiritual relationships. Such contrast suggests that integrating cultural formulation interviews or ethnographic assessment tools into PTSD diagnostics could enhance cross-cultural validity.

In the Democratic Republic of Congo, particularly in Kinshasa, mental distress is frequently attributed to supernatural causes such as witchcraft or ancestral spirits rather than medical conditions (Wiel and Slegh, 2024). Generational differences have also been observed: older participants often endorsed beliefs in demonic possession and witchcraft, while younger cohorts showed more openness to medical explanations (Slewa-Younan et al., 2022). In the Sahel region, including parts of Mali and Senegal, Islamic spiritual healing traditions similarly frame trauma as a form of spiritual imbalance. Practices such as dhikr (remembrance of God) and Quranic recitation have been shown to promote mental well-being and alleviate conditions like anxiety and addiction (Zahir and Qoronfleh, 2025). In Pentecostal and charismatic Christian settings, particularly in South Africa, experiences such as insomnia, visions, or dissociative states are often understood through spiritual lenses, attributed to demonic interference or the need for deliverance rather than conceptualized as clinical manifestations of PTSD (Captari and Worthington, 2024). Similarly, traditional healers and urban spiritual practitioners, especially in cities like Johannesburg, frequently interpret mental distress as arising from supernatural causes, including ancestral displeasure or witchcraft, and prescribe rituals aimed at restoring spiritual balance (Galvin et al., 2023). Taken together, these culturally embedded explanatory models underline the importance of complementing DSM-5's cultural formulation framework with deeper engagement in anthropological and local contexts.

### 4.2 Cultural beliefs and help-Seeking

Cultural beliefs in fate, witchcraft, and sorcery diverge from DSM-5's psychological interpretations, which attribute PTSD symptoms to biopsychological processes (American Psychiatric Association, 2016). Because illness is frequently conceptualized as spiritually driven, some individuals may prefer ritual or spiritual healing over psychological care. Instead, they turn to traditional rituals and spiritual healing, reinforcing the view that PTSD is a spiritual disorder characterized by somatic symptoms, persecution dreams, aggression, and muteness (Mavinga et al., 2022).

This preference does not necessarily reflect rejection of biomedical models but rather an attempt to restore moral and relational order within the community. As such, help-seeking behaviors are embedded in moral economies of care rather than purely medical logics.

In Sub-Sahara African societies, emotional distress is often linked to physical symptoms, and when medical treatments fail, individuals seek healing from God and ancestors (Knettel et al., 2020; Koudou et al., 2019; Pireyre et al., 2019; Van der Kolk et al., 2016). While psychological care is increasingly available in urban settings, cultural ceremonies remain central to many communities in addressing PTSD (Brohm, 2017; Pireyre et al., 2019). Recognizing these perspectives is crucial for developing culturally relevant PTSD treatments.

### 4.3 Healing rituals in Sub-Saharan Africa

Rituals play a fundamental role in African societies by maintaining communal bonds and restoring unity (Brohm, 2017; Kaminer et al., 2018; Mavinga et al., 2022; Pireyre et al., 2019). After traumatic events, victims often seek assistance from traditional healers (ngangas) rather than psychologists, even when the latter share their cultural background. Even when psychological treatments are acknowledged as effective, many individuals supplement them with spiritual and ritualistic healing (Desjardins et al., 2022).

This reliance on traditional healing is linked to the perception that trauma ruptures social and cosmological order rather than individual mental stability. Many African cultures view trauma symptoms as spiritually caused, prompting individuals to engage in prayer and purification rituals. These ceremonies, particularly those addressing sexual violence and misfortune, aim to cleanse both the individual and their community (Mouanga et al., 2018). Hence, collective ritual healing performs both a therapeutic and a sociopolitical function by re-establishing moral equilibrium. The collective approach to trauma treatment underscores the importance of culturally sensitive interventions.

### 4.4 Study strengths and limitations

Conducting a scoping review comparing DSM-5 and Sub-Saharan Africa explanatory models of PTSD presents both strengths and limitations.

Strengths:

1. Comprehensive mapping: Scoping reviews offer a broad overview of PTSD research, highlighting knowledge gaps and research opportunities.
2. Cultural sensitivity: Examining PTSD through both DSM-5 and Sub-Saharan Africa perspectives fosters more effective, culturally appropriate mental health care.
3. Integrative care framework: Identifying overlaps between DSM-5 and African models helps develop integrative mental health care approaches, improving trauma interventions.

Limitations:

- Variability in data quality: The inclusion of studies with diverse methodologies complicates direct comparisons and limits definitive conclusions.
- Limited depth of analysis: While scoping reviews provide broad insights, they may lack the depth of systematic reviews, potentially overlooking cultural nuances.
- Potential for cultural bias: Interpretation of data across cultural contexts may be biased by researchers' perspectives, emphasizing the need for local stakeholder involvement.
- Methodological constraints: Inconsistent reporting across included studies, relatively narrow inclusion criteria, and limited reproducibility of the search strategy further restrict generalizability.

Despite these limitations, this scoping review provides valuable insights into how PTSD is understood and treated in sub-Saharan Africa. Future research should prioritize participatory designs and community co-analysis to ensure that indigenous voices shape conceptual frameworks and intervention models. Enhancing cultural collaboration can further strengthen the validity of findings.

### 4.5 Clinical practice recommendations

This review underscores how traditional beliefs shape PTSD symptoms in African populations and highlights the necessity of culturally appropriate care. Addressing PTSD in sub-Saharan Africa requires integrating cultural models into psychological interventions through a multidisciplinary approach. Importantly, the following recommendations are provided as illustrative examples from broader literature and not as findings derived from the included studies. Together, these recommendations reflect an emerging consensus in global mental health that sustainable trauma care in post-conflict Africa must bridge biomedical, spiritual, and community-based paradigms.

#### 4.5.1 Culturally adapted psychotherapy

Adapting PTSD interventions like Cognitive Behavioral Therapy (CBT) and Narrative Exposure Therapy (NET) to cultural contexts enhances therapy effectiveness. Integrating storytelling, metaphors, and oral traditions ensures therapy aligns with local experiences. For example, in Rwanda, community-based NET incorporating local storytelling successfully helped trauma survivors reconstruct their experiences (Lesley et al., 2024). Culturally adapted interventions, particularly those incorporating elements of traditional healing, may contribute to effective PTSD symptom reduction. A meta-analysis found that blending traditional and modern therapies yielded significant positive effects (Arundell et al., 2021). Recognizing cultural values allows mental health professionals to develop effective, personalized treatments (Marques et al., 2021). Prioritizing culturally grounded research, is therefore essential for tailoring PTSD diagnosis and treatment to sub-Saharan African populations.

#### 4.5.2 Validation of diagnostic tools

Assessing PTSD diagnostic instruments' applicability in sub-Saharan Africa is vital. Western-developed tools like the PTSD Checklist for DSM-5 (PCL-5) may not capture culturally specific trauma expressions. A study validating the Shona PCL-5 in Zimbabwe emphasized the need for cultural adaptation (Verhey et al., 2018). Similarly, research on the Impact of Event Scale-Revised (IES-R) found that higher cut-off points were necessary in African populations (Abas et al., 2023). These examples highlight how contextually validated tools can improve diagnostic accuracy and support the development of more culturally appropriate interventions.

#### 4.5.3 Collaboration with traditional healers and religious leaders

Training traditional healers, pastors, and community elders in trauma-informed care fosters a collaborative referral system. This integration bridges cultural beliefs and psychological care, improving treatment outcomes. For instance, in Uganda, partnerships between healers and psychologists have strengthened mental health services for war survivors (Sekagya et al., 2024).

#### 4.5.4 Feminist perspectives

Feminist scholars adds further depth in understanding trauma in Africa. Some analyses suggest that psychiatric discourse may risk reinforcing patriarchal power relations if it pathologizes women's suffering without addressing its structural causes (Lokot et al., 2024). For example, gender-based violence and practices such as female genital mutilation/cutting are embedded in cultural contexts that deeply shape trauma experiences, yet are often underexplored in global psychiatric nosologies (Tammary and Manasi, 2023). Research on help-seeking among women in Sub-Saharan contexts also shows how stigma and cultural blame frequently silence survivors and prevent access to care (O'Mullan et al., 2024; Owusu-Antwi et al., 2024).

#### 4.5.5 Community-based interventions and group therapy

Establishing group therapy in familiar settings, such as churches and community centers, reduces stigma and promotes collective healing. Cultural elements like rituals, music, and dance facilitate emotional expression and recovery. As an illustration, in Sierra Leone, drumming therapy has helped ex-child soldiers process trauma, demonstrating the benefits of integrating traditional practices into mental health care (Ngobili, 2024).

#### 4.5.6 Integrating a psychomotricity approach

Understanding the connection between psychological processes and motor functions is crucial in sub-Saharan Africa (Rousseau and Cook-Cottone, 2018). Psychomotricity aligns with traditional African healing practices, emphasizing the mind-body-spirit connection (Erlank and Williams, 2019). Housseini-Houy et al. (2022) stress the role of psychomotricity in cultural transmission and trauma recovery. One recent study in DRC has also reported positive impacts of music therapy with songwriting for mental health of vulnerable populations in conflict-ridden settings (Alexandre et al., 2025). Taken together, these findings suggest that integrating psychomotricity, narrative, and spiritual dimensions could form the basis of culturally grounded trauma rehabilitation models in Sub-Saharan Africa.

### 4.6 Broader implications

This review contributes to the decolonization of trauma studies by challenging the universal applicability of Western diagnostic frameworks and advocating for pluralistic, context-sensitive understandings of suffering. Recognizing the coexistence of biomedical and spiritual logics in African trauma healing may inform global psychiatry to adopt a more inclusive epistemology one that values relational and communal dimensions of recovery.

## 5 Conclusion

This scoping review underscores the divergence between DSM-5 and sub-Saharan African explanatory models of PTSD, while also identifying points of convergence that support more integrative approaches to trauma care. Situating PTSD symptoms within cultural frameworks not only validates lived experiences but also helps engage local support systems that are central to healing. In many sub-Saharan contexts, cleansing rituals following traumatic events such as rape are intended to remove perceived impurities and restore balance. While these practices may foster personal and communal recovery, the current evidence base is insufficient to justify their direct integration into clinical interventions. Clinicians are therefore encouraged to practice cultural competence, acknowledging and respecting cultural and spiritual frameworks while maintaining evidence-based standards. Such an approach can strengthen therapeutic alliances, affirms patients' realities, and advances culturally responsive mental health care.

## Data Availability

The original contributions presented in the study are included in the article/supplementary material, further inquiries can be directed to the corresponding author.
